# Genome Assembly and Genome Annotation of *Leishmania martiniquensis* Isolated from a Leishmaniasis Patient in Thailand

**DOI:** 10.1155/2022/8768574

**Published:** 2022-03-22

**Authors:** Songtham Anuntakarun, Atchara Phumee, Vorthon Sawaswong, Kesmanee Praianantathavorn, Witthaya Poomipak, Rungrat Jitvaropas, Padet Siriyasatien, Sunchai Payungporn

**Affiliations:** ^1^Program in Bioinformatics and Computational Biology, Graduate School, Chulalongkorn University, Bangkok, Thailand; ^2^Department of Medical Technology, School of Allied Health Sciences, Walailak University, Nakhon Si Thammarat, Thailand; ^3^Department of Biochemistry, Faculty of Medicine, Chulalongkorn University, Bangkok, Thailand; ^4^Research Affairs, Faculty of Medicine, Chulalongkorn University, Bangkok, Thailand; ^5^Division of Biochemistry, Department of Preclinical Science, Faculty of Medicine, Thammasat University, Pathum Thani, Thailand; ^6^Vector Biology and Vector Borne Disease Research Unit, Department of Parasitology, Faculty of Medicine, Chulalongkorn University, Bangkok, Thailand; ^7^Research Unit of Systems Microbiology, Chulalongkorn University, Bangkok, Thailand

## Abstract

Leishmaniasis is a parasitic disease caused by *Leishmania* spp. with worldwide distribution. Autochthonous leishmaniasis has been reported to result from the infection by *Leishmania martiniquensis* in Thailand. This species was isolated in culture and subjected to high-throughput whole-genome sequencing. A total of 30.8 Mb in 36 chromosomes of the whole genome was assembled, annotated, and characterized. The *L. martiniquensis* under study was shown to segregate into the same clade and thus closely related to the previously identified *L. martiniquensis* (LU_Lmar_1.0), as determined by phylogenetic analysis of their genomic sequences along with those of representative kinetoplastid species. The total number of open reading frames genomewide predicts 8,209 protein-coding genes, of which 359 are putative virulence factors, including two previously known, e.g., cysteine proteinase C and superoxide dismutase B1. The results obtained from this study will be useful for further annotation and comparison with other *Leishmania martiniquensis* in the future.

## 1. Introduction

*Leishmania* species are members of the Class Kinetoplastea, Order Trypanosomatida. They are intracellular protozoa that are transmitted through vertebrate hosts by infected female phlebotomine sandflies. There are three major clinical presentations of the disease including cutaneous leishmaniasis (CL), mucocutaneous leishmaniasis (MCL), and visceral leishmaniasis (VL). Symptoms of CL occur on the skin with wet or dry ulcers that are usually painless and localized lesions, while MCL produces sores on mucosal surfaces, especially the nose, mouth, or throat. VL is the most severe form which occurs in internal organs including the spleen, liver, lymph nodes, and bone marrow. Recently, a novel subgenus *Mundinia* of *Leishmania* parasites has been described, which consists of *L. martiniquensis*, *L. orientalis* n. sp. (previously called *L. siamensis*), *L. enriettii*, and *L. macropodum* (previously called “*Leishmania sp.* AM-2004”) [1–4]. Only *L. martiniquensis* and *L. orientalis* n. sp. have been described as causative agents of leishmaniasis in humans [1, 5, 6].

In Thailand, autochthonous leishmaniasis is caused by *L. martiniquensis* and *L. orientalis n.* sp. (*L. siamensis*). The prevalence of *L. martiniquensis* cases in Thailand have dramatically increased in recent years [7, 8]. Indigenous leishmaniasis cases in Thailand caused by *L. martiniquensis* and *L. orientalis* n. sp. were presented with CL, VL, and overlapping CL and VL. Most of the cases were found in immunocompromised patients especially those with AIDS, and these patients also present a poor response to medical treatment. Amphotericin B is the only antileishmanial agent available for the treatment of indigenous leishmaniasis in Thailand. Cases of relapsed leishmaniasis caused by *L. martiniquensis* were found after receiving amphotericin B treatment [9]. Therefore, the whole-genome sequencing of *L. martiniquensis* would be useful for the understanding of virulence factor genes and interpretation of clinical severity and manifestations.

There have been many studies of the *Leishmania* genome in various species based on next-generation sequencing during the past few years [10, 11]. Currently, it is known that there are virulence factor genes in protozoans including *Leishmania* species. These genes are related to parasite survival and infection in the host cells. For example, proteins such as chaperones and endoribonuclease L-PSP can improve the survival rate of the parasite. In addition, some enzymes are related to migration to facilitate infection of host cells [12]. Particular proteinases, e.g., cysteine-proteinases, metalloproteinases, and serine-proteinases, are also known as virulence factors in *Leishmania* spp. Moreover, they have a wide range of biological roles, including the mechanism of infection [13].

In this study, the *L. martiniquensis* genome was sequenced, assembled, and explored, providing a genetic resource for future exploration of the biology of a clinical isolate of under study species. Subsequently, virulence factor genes in this genome were predicted and analyzed. The candidate virulence factor genes will be validated in further studies.

## 2. Materials and Methods

### 2.1. Ethics Statement

This study was approved by the Institutional Review Board of the Faculty of Medicine, Chulalongkorn University, Bangkok, Thailand (COA No. 768/2012). Patients were not involved in this study.

### 2.2. Promastigote of *L. martiniquensis* Culture

The promastigotes of *L. martiniquensis* (MHOM/TH/2011/CU1) were isolated from the bone marrow of an overlapping CL and VL patient in Songkhla province, Southern Thailand, in 2011 [14–17]. The promastigotes were cultured in Schneider's Insect Medium (Sigma-Aldrich, Missouri, USA) at a pH of 6.7 supplemented with 10% heat-inactivated fetal bovine serum, 100 U/ml penicillin, and 100 *μ*g/ml streptomycin. The promastigotes were incubated at 25 ± 2°C in an incubator and inspected for parasite viability every day under an inverted microscope (Olympus, Tokyo, Japan).

### 2.3. DNA Extraction

The *L. martiniquensis* promastigotes (10^6^ parasites/ml) were washed with 1x phosphate-buffered saline (PBS) three times (Sigma-Aldrich, Missouri, USA) and centrifuged at 11,000 × g for 10 min. The sample was ground in lysis buffer and used for DNA extraction by using an Invisorb Spin Tissue Mini Kit (STRATEC Molecular, Berlin, Germany), following the manufacturer's instructions. The DNA concentration and purity were quantified by a Qubit 2.0 Fluorometer (Invitrogen, Massachusetts, USA). The extracted DNA samples were used for sequencing immediately, and the rest of the samples were stored at –80°C.

### 2.4. Library Preparation and High-Throughput Sequencing

DNA (1 *μ*g) was fragmented by using a Covaris M220 focused-ultrasonicator (Covaris, Brighton, UK) with a 20% duty factor, 50 units of peak incident power (W), and 200 cycles per burst for 150 seconds. Then, the fragmented DNA was used for DNA library preparation based on the TruSeq DNA LT Sample Prep Kit (Illumina, California, USA) following the manufacturer's instructions. The DNA library was cleaned up, and the size was selected by AMPure XP beads (Beckman Coulter, USA). The concentration of library DNA was measured by using the KAPA Library Quantification Kit (Kapa Biosystems, Massachusetts, USA). The DNA library was diluted to 6 pM and then paired-end sequenced (2 × 150 bp) based on the MiSeq platform (Illumina, California, USA) by using MiSeq Reagent Kits V2 (300 cycles) according to the standard protocol.

### 2.5. Quality Filter and Genome Assembly

FASTQ files with 150 bp paired-end reads were checked for the quality of sequences by FastQC [18]. Trimmomatic version 0.39 [19] was used to trim and remove low-quality reads using default parameters. *De novo* assembly was performed using SPAdes version 3.12.0 [20]. The scaffold sequences from the previous step were used to align with the *Leishmania martiniquensis* genome from the NCBI database (accession number CM030396.1–CM030431.1 for chromosome 1–36) [21] using the Artemis comparison tool (ACT) [22]. In addition, the Illumina reads were mapped into the assembled scaffolds using BWA version 0.7.16a [23].

### 2.6. Gene Prediction and Functional Annotation

AUGUSTUS (Galaxy version 3.3.3) [24] was used to predict genes in the *L. martiniquensis* genome. In this work, the *Leishmania tarentolae* model organism was used in the species parameter for the prediction of gene locations and protein-coding genes. Putative protein-coding sequences from AUGUSTUS were performed in the functional annotation. The EggNOG-mapper version 2 [25] (default parameters) was used to predict functional annotation against EggNOG 5.0 [26]. This database contains functional information from many sources including a Cluster of Orthologous Groups of proteins (COGs) [27], KEGG pathway [28], and GO annotation [29].

### 2.7. Prediction of Virulence Factor Genes

Putative protein-coding sequences were analyzed by blastP with the protozoa virulence protein database (ProtVirDB) [30] and pathogen host interaction database (PHI-base) [31] for predicting candidate virulence factor proteins and interaction between hosts and pathogens, respectively. In this study, the criteria for the determination of candidate virulence sequences were an *e*-value of 10*e*^−5^ or less. For proteinase gene analysis, proteinase genes of *L. martiniquensis* were predicted using sequences from the previous report [13] as a reference.

### 2.8. Phylogenetic Tree Analysis

OrthoFinder version 2.5.2 [32] with default parameters was used for finding single-copy orthologous genes and alignment of single-copy orthologous genes. In this study, the protein sequence dataset from various species including *Trypanosoma brucei* TREU927 (GCF_000002445.2), *Trypanosoma vivax* Y486 (CA_000227375.1), *Trypanosoma grayi* (GCF_000691245.1), *Trypanosoma cruzi* strain CL Brener (GCF_000209065.1), *Trypanosoma rangeli* (GCF_003719475.1), *Phytomonas* sp. isolate EM1 (GCA_000582765.1), *Leptomonas seymouri* (GCA_001299535.1), *Leptomonas pyrrhocoris* (GCF_001293395.1), *Leishmania enriettii* (GCA_017916305.1), *Leishmania martiniquensis* (GCA_017916325.1), *Leishmania tarentolae* (GCA_009731335.1), *Leishmania mexicana* MHOM/GT/2001/U1103 (GCF_000234665.1), *Leishmania major* strain Friedlin (GCF_000002725.1), *Leishmania donovani* (GCF_000227135.1), *Leishmania infantum* JPCM5 (GCF_000002875.1), *Leishmania panamensis* (GCF_000755165.1), *Leishmania braziliensis* MHOM/BR/75/M2904 (GCF_000002845.1), and our *Leishmania martiniquensis* (MHOM/TH/2011/CU1) were used as input of the OrthoFinder tool. The Newick format of phylogenetic tree from OrthoFinder was visualized using Interactive Tree Of Life (iTOL) (https://itol.embl.de/) [33].

### 2.9. Comparison of *L. martiniquensis* Genome with Other Leishmania Species

The analysis of the percentage identity of *Leishmania* chromosomes was performed on representative *Leishmania* species including *Leishmania major* strain Friedlin (GCF_000002725.1), *Leishmania infantum* JPCM5 (GCF_000002875.1), *Leishmania donovani* (GCF_000227135.1), *Leishmania mexicana* MHOM/GT/2001/U1103 (GCF_000234665.1), *and Leishmania martiniquensis* MHOM/TH/2012/LSCM1 (LU_Lmar_1.0) (GCA_017916325.1) using Clustal Omega version 1.2.4 with default parameters [34].

## 3. Results

### 3.1. Genome Characteristics of *L. martiniquensis* Genome

Paired-end FASTQ files were used for *de novo* assembly using SPAdes. After assembly, there were 6,939 scaffolds with N50 63,362 bp. The percentage of mapped reads to assembled scaffolds between *L. martiniquensis* in this study and *L. martiniquensis* (LU_Lmar_1.0) was 82.36%.

The statistics of *L. martiniquensis* data are shown in Table 1. After the gene prediction step, there were 8,209 protein-coding genes in the final assembly of chromosome 1 to chromosome 36. The chromosome size ranges from 0.24 to 2.8 Mb. The existence of regions in the genome with large variations in the CG content may be caused by over- or underfragmentation during the library construction. The *L. martiniquensis* genome had an average GC content of 59.77%.

### 3.2. Comparison of *L. martiniquensis* with Other *Leishmania* Species

**Figure 1:**
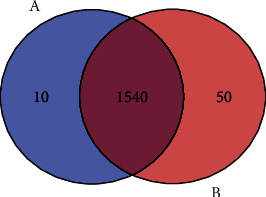
Venn diagram analysis of genes between *Leishmania martiniquensis* (LU_Lmar_1.0) genome from the NCBI database (a) and *Leishmania martiniquensis* from our study (b).

The genome (36 chromosomes) of *L. martiniquensis* was compared with other *Leishmania* species including *L. infantum*, *L. donovani*, *L. braziliensis*, *L. major* strain Friedlin, *L. mexicana*, and *L. martiniquensis*. In addition, the genome of *L. martiniquensis* in this study was closely related to *L. martiniquensis* (LU_Lmar_1.0). Genome coverage between *L. martiniquensis* in this study and *L. martiniquensis* (LU_Lmar_1.0) was 95.18%. The Venn diagram analysis in Figure 1 shows that 10 genes and 50 genes were found in only *L. martiniquensis* (LU_Lmar_1.0) genome and our *L. martiniquensis*, respectively. In addition, the descriptions of genes are shown in Supplementary Material 3. The result of nucleotide identity is summarized in Supplementary Table 1. The COG functional category in *L. martiniquensis* was compared with other *Leishmania* spp. including *L. infantum*, *L. donovani*, *L. braziliensis*, *L. major*, and *L. mexicana*. Our results showed that *L. martiniquensis* genes occupied similar functional roles to those in other *Leishmania* spp. (Supplementary Table 2). The KEGG pathway analysis and GO annotation are represented in Supplementary Figure 1. In the KEGG pathway analysis (Supplementary Figure 1A), the top three pathways include ribosome, metabolic pathways, and RNA polymerase. Functional annotation is the process of collecting information about the function of genes. Gene Ontology (GO) is the most widespread and extensive functional annotation for gene and protein sequences. There are three categories of terms in Gene Ontology. First, the molecular function comprises the molecular activities of individual gene products. Second, the cellular component comprises the region of active gene products. Third, the biological process comprises the processes and the pathways in which the activity of gene products is involved. The result of GO analysis in Supplementary Figures 1B-1D shows that the top three molecular functions were structural constituent of ribosome, poly (A) RNA-binding, and DNA-directed RNA polymerase activity. The top three cellular component functions were cytosolic large ribosomal subunit, motile cilium, and intraciliary transport particle B. The top three biological process functions were translation, rRNA processing, and ribosomal large subunit assembly.

### 3.3. Virulence Factor Gene Analysis

Predicted proteins for the protein-coding genes predicted by AUGUSTUS were analyzed by blastP with the protozoa virulence protein database (ProtVirDB) using the criteria of *e*-value <10*e*^−5^. A total of 359 genes were found as candidate virulence factor genes. These genes were then analyzed for COG functional annotation. The top 3 COG functions were signal transduction mechanism, carbohydrate transport and metabolism, and intracellular trafficking, secretion, and vesicular transport, while the remaining COG functions are shown in Supplementary Figure 2. The annotation lists of the 359 genes from ProtVirDB are shown in Supplementary Material 1. Moreover, forty-three predicted protein sequences that passed the criteria from blastP with PHI-base were related with *Homo sapiens* organisms. The annotation lists of the 43 genes from the PHI-base are shown in Supplementary Material 2. However, the predicted virulence factor gene should be validated in further study.

### 3.4. Phylogenetic Tree Analysis

**Figure 2:**
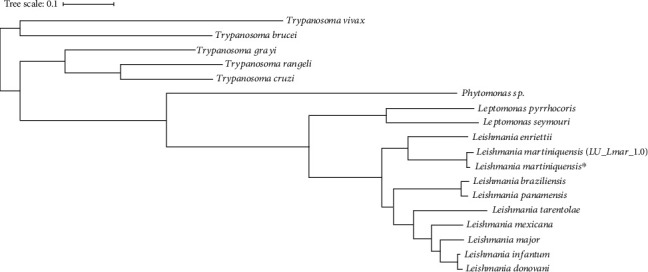
The phylogenetic analysis based on 17 single-copy shared orthologous genes obtained from OrthoFinder tool. The star symbol represents our *Leishmania martiniquensis* (MHOM/TH/2011/CU1).

The concatenated protein sequences of 17 single-copy orthologous genes were used to create a phylogenetic tree. In Figure 2, the phylogenetic tree indicates that *L. martiniquensis* is related to *Leishmania* spp. Moreover, the outgroup including *Trypanosoma brucei* TREU927, *Trypanosoma vivax* Y486, *Trypanosoma grayi*, *Trypanosoma cruzi* strain CL Brener, and *Trypanosoma rangeli* is a more distinctly related group of the *Leishmania* species. The result suggests that *L. martiniquensis* in this study is closely related to *L. martiniquensis* (LU_Lmar_1.0) published in April 2021 on the NCBI website.

## 4. Discussion

In this study, genome assembly and gene prediction of *Leishmania martiniquensis* were performed. The results showed that the COG functional category of *L. martiniquensis* was similar to other *Leishmania* species. However, there was a slight difference in the number of genes in each functional group. The importance of parasite virulence factors has become apparent in recent years [35]. The variability of virulence factor genes within the *Leishmania* species is largely unknown. In our virulence factor gene prediction of *L. martiniquensis*, the result showed that 359 candidate virulence factor genes were found in *L. martiniquensis*. Some of these genes are discussed below.

Heat shock proteins are intracellular molecules of varying molecular weights. They are a large family of molecular chaperones. The role of this protein is maturation, degradation, and refolding [36]. They also play an important role in immune biological functions, especially in *hsp70*. There was a report which showed that *hsp70* induces dendritic cells to generate proinflammatory cytokines [37] and is related to the enhancement of adaptive immunity [38]. In our results, the *hsp70* protein-coding gene in 359 candidate virulence factor genes was found. This gene might be related to the infection of host cells.

Proteinases are enzymes that hydrolyze peptide bonds in proteins and participate in a wide range of biological functions, including the process of infection [13]. There are many classes of proteinase based on catalytic domains [39]. There are only three classes, including aspartyl-, metallo-, and cysteine-proteinase, which have been extensively studied in *Leishmania* organisms [40, 41]. In a previous review, cysteine proteases were considered to play a crucial role in the pathogenesis of other parasitic protozoan infections [42]. *CPA*, *CPB*, and *CPC* genes in a group of cysteine proteases have been widely studied in *Leishmania* species. In our analysis result, *CPC* genes were found in *L. martiniquensis*. *CPC* plays a potential role in resisting parasite killing by macrophages [43].

In addition, superoxide dismutase gene (*SODB1*) was found only in our genome when compared with *L. martiniquensis* (LU_Lmar_1.0). There is a report that *SODB1* is required for *Leishmania major* pathogenicity in mice and persistence in macrophages [44]. Superoxide dismutases (SODs) are metalloenzymes that convert superoxide to oxygen and hydrogen peroxide in the antioxidant defense system. Nickel (Ni), manganese (Mn), iron (Fe), or copper and zinc (Cu/Zn) are cofactors of superoxide dismutases [45, 46].

The phylogenetic analysis showed that our *L. martiniquensis* is closely related to the previously reported *L. martiniquensis* (LU_Lmar_1.0) reference genome in the NCBI database. Comparative genomics of *L.* (*Mundinia*) *martiniquensis* was reported in 2019 [47]. This research undertaken here reported genomes of *L.* (*Mundinia*) *martiniquensis* with a protein dataset.

## 5. Conclusions

In this study, *L. martiniquensis* genomic DNA was successfully sequenced and was assembled into 6939 scaffolds. A total of 30,784,469 bp in 36 chromosomes of the *L. martiniquensis* genome were analyzed. The analysis results showed that the general features of *L. martiniquensis* were similar to other *Leishmania* species, including chromosome sizes, the number of protein-coding genes, and the GC contents. In addition, the results of COG functional annotation were shown to be similar to other *Leishmania* species. In the virulence factor gene prediction result, 359 potential candidate virulence factor genes were found in this study. Most predicted virulence factor genes were related to RNA processing and modification function. However, candidate potential virulence factor genes should be validated in a further study using experimental study.

## Figures and Tables

**Table 1:** Statistics of *L. martiniquensis* data and *de novo* assembly.

Genome features of *L. martiniquensis*	
Read length (bp)	150
Raw reads	26,205,720
Q30 reads	23,836,943
Number of scaffolds	6,939
N50 (bp)	63,362
Number of protein-coding genes	8,209
Illumina read mapping to the assembled scaffolds (%)	82.36

## Data Availability

All data analyzed during this study are included in this article. In addition, the DNA sequences were deposited in Sequence Read Archive (SRA) data of the NCBI server (BioProject ID PRJNA674467).

## Supplementary Materials

Supplementary 1 Supplementary Material 1: the annotation lists of the candidate predicted virulence factor genes from ProtVirDB.

Supplementary 2 Supplementary Material 2: the annotation lists of the candidate predicted virulence factor genes from PHI-base.

Supplementary 3 Supplementary Material 3: the annotation lists of the unique genes between *L. martiniquensis* (LU_Lmar_1.0) and our *L. martiniquensis* genome.

Supplementary 4 Supplementary Table 1: comparison of percent identity in *Leishmania* spp. including *L. infantum*, *L. donovani*, *L. major Friedlin*, *L. Mexicana*, *L. braziliensis*, *L. martiniquensis*, and our *L. martiniquensis*. Supplementary Table 2: comparison of functional category of putative protein-coding genes in *Leishmania spp.* genome. The alphabet A-G represent names of *Leishmania* species including *L. martiniquensis* (A), *L. infantum* (B), *L. donovani* (C), *L. braziliensis* (D), *L. major* strain Friedlin (E), *L. mexicana* (F), and *L. martiniquensis* (LU_Lmar_1.0) (G).

Supplementary 5 Supplementary Figure 1: the functional annotation of *L. martiniquensis*. Supplementary Figure 2: the COG functional analysis of candidate virulence factor protein-coding genes.

## References

[1] Jariyapan N., Daroontum T., Jaiwong K. (2018). Leishmania (Mundinia) orientalis n. sp. (Trypanosomatidae), a parasite from Thailand responsible for localised cutaneous leishmaniasis. *Parasites and Vectors*.

[2] Espinosa O. A., Serrano M. G., Camargo E. P., Teixeira M. M. G., Shaw J. J. (2018). An appraisal of the taxonomy and nomenclature of trypanosomatids presently classified as Leishmania and Endotrypanum. *Parasitology*.

[3] Barratt J., Kaufer A., Peters B. (2017). Isolation of novel trypanosomatid, Zelonia australiensis sp. nov. (Kinetoplastida: Trypanosomatidae) provides support for a Gondwanan origin of dixenous parasitism in the Leishmaniinae. *PLoS Neglected Tropical Diseases*.

[4] Sukmee T., Siripattanapipong S., Mungthin M. (2008). A suspected new species of _Leishmania_ , the causative agent of visceral leishmaniasis in a Thai patient. *International Journal for Parasitology*.

[5] Chiewchanvit S., Tovanabutra N., Jariyapan N. (2015). Chronic generalized fibrotic skin lesions from disseminated leishmaniasis caused by Leishmania martiniquensis in two patients from northern Thailand infected with HIV. *The British Journal of Dermatology*.

[6] Pothirat T., Tantiworawit A., Chaiwarith R. (2014). First isolation of Leishmania from northern Thailand: case report, identification as Leishmania martiniquensis and phylogenetic position within the Leishmania enriettii complex. *PLoS neglected tropical diseases*.

[7] Siriyasatien P., Chusri S., Kraivichian K. (2016). Early detection of novel Leishmania species DNA in the saliva of two HIV-infected patients. *BMC Infectious Diseases*.

[8] Phumee A., Chusri S., Kraivichian K. (2014). Multiple cutaneous nodules in an HIV-infected patient. *PLoS Neglected Tropical Diseases*.

[9] Phumee A., Jariyapan N., Chusri S. (2020). Determination of anti-leishmanial drugs efficacy against _Leishmania martiniquensis_ using a colorimetric assay. *Parasite Epidemiology and Control*.

[10] Lypaczewski P., Hoshizaki J., Zhang W. W. (2018). A complete _Leishmania donovani_ reference genome identifies novel genetic variations associated with virulence. *Scientific Reports*.

[11] Coughlan S., Taylor A. S., Feane E. (2018). Leishmania naiffiandLeishmania guyanensisreference genomes highlight genome structure and gene evolution in theVianniasubgenus. *Royal Society open science*.

[12] Da Fonseca Pires S., Fialho L. C., Silva S. O. (2014). Identification of virulence factors in Leishmania infantum strains by a proteomic approach. *Journal of Proteome Research*.

[13] Silva-Almeida M., Pereira B. A. S., Ribeiro-Guimarães M. L., Alves C. R. (2012). Proteinases as virulence factors in Leishmania spp. infection in mammals. *Parasites and Vectors*.

[14] Chusri S., Hortiwakul T., Silpapojakul K., Siriyasatien P. (2012). Consecutive cutaneous and visceral leishmaniasis manifestations involving a novel Leishmania species in two HIV patients in Thailand. *The American Journal of Tropical Medicine and Hygiene*.

[15] Phumee A., Kraivichian K., Chusri S. (2013). Detection of Leishmania siamensis DNA in saliva by polymerase chain reaction. *The American Journal of Tropical Medicine and Hygiene*.

[16] Becvar T., Vojtkova B., Siriyasatien P. (2021). Experimental transmission of Leishmania (Mundinia) parasites by biting midges (Diptera: Ceratopogonidae). *PLoS Pathogens*.

[17] Leelayoova S., Siripattanapipong S., Hitakarun A. (2013). Multilocus characterization and phylogenetic analysis of Leishmania siamensis isolated from autochthonous visceral leishmaniasis cases, southern Thailand. *BMC Microbiology*.

[18] Andrews S. (2010). *Fast QC: a quality control tool for high throughput sequence data*.

[19] Bolger A. M., Lohse M., Usadel B. (2014). Trimmomatic: a flexible trimmer for Illumina sequence data. *Bioinformatics*.

[20] Bankevich A., Nurk S., Antipov D. (2012). SPAdes: a new genome assembly algorithm and its applications to single-cell sequencing. *Journal of Computational Biology*.

[21] Almutairi H., Urbaniak M. D., Bates M. D. (2021). Chromosome-scale assembly of the complete genome sequence of Leishmania (Mundinia) martiniquensis, isolate LSCM1, strain LV760. *Microbiology Resource Announcements*.

[22] Carver T. J., Rutherford K. M., Berriman M., Rajandream M. A., Barrell B. G., Parkhill J. (2005). ACT: the Artemis comparison tool. *Bioinformatics*.

[23] Li H., Durbin R. (2009). Fast and accurate short read alignment with Burrows-Wheeler transform. *Bioinformatics*.

[24] Stanke M., Morgenstern B. (2005). AUGUSTUS: a web server for gene prediction in eukaryotes that allows user-defined constraints. *Nucleic Acids Research*.

[25] Cantalapiedra C. P., Hernández-Plaza A., Letunic I., Huerta-Cepas J. (2021). eggNOG-mapper v2: functional annotation, orthology assignments, and domain prediction at the metagenomic scale. *BioRxiv*.

[26] Huerta-Cepas J., Szklarczyk D., Heller D. (2019). EggNOG 5.0: a hierarchical, functionally and phylogenetically annotated orthology resource based on 5090 organisms and 2502 viruses. *Nucleic acids research*.

[27] Galperin M. Y., Makarova K. S., Wolf Y. I., Koonin E. V. (2015). Expanded microbial genome coverage and improved protein family annotation in the COG database. *Nucleic Acids Research*.

[28] Kanehisa M., Furumichi M., Tanabe M., Sato Y., Morishima K. (2017). KEGG: new perspectives on genomes, pathways, diseases and drugs. *Nucleic Acids Research*.

[29] Harris M. A., Clark J., Ireland A. (2004). The Gene Oncology (GO) database and informatics resource. *Nucleic acids research*.

[30] Ramana J., Gupta D. (2009). ProtVirDB: a database of protozoan virulent proteins. *Bioinformatics*.

[31] Urban M., Cuzick A., Rutherford K. (2017). PHI-base: a new interface and further additions for the multi-species pathogen-host interactions database. *Nucleic Acids Research*.

[32] Emms D. M., Kelly S. (2019). OrthoFinder: phylogenetic orthology inference for comparative genomics. *Genome Biology*.

[33] Letunic I., Bork P. (2021). Interactive tree of life (iTOL) v5: an online tool for phylogenetic tree display and annotation. *Nucleic Acids Research*.

[34] Sievers F., Wilm A., Dineen D. (2011). Fast, scalable generation of high-quality protein multiple sequence alignments using Clustal Omega. *Molecular systems biology*.

[35] Urrea D. A., Duitama J., Imamura H. (2018). Genomic analysis of Colombian _Leishmania panamensis_ strains with different level of virulence. *Scientific Reports*.

[36] Miller D. J., Fort P. E. (2018). Heat shock proteins regulatory role in neurodevelopment. *Frontiers in Neuroscience*.

[37] Kuppner M. C., Gastpar R., Gelwer S. (2001). The role of heat shock protein (hsp70) in dendritic cell maturation: Hsp70 induces the maturation of immature dendritic cells but reduces DC differentiation from monocyte precursors. *European Journal of Immunology*.

[38] MacAry P. A., Javid B., Floto R. A. (2004). HSP70 peptide binding mutants separate antigen delivery from dendritic cell stimulation. *Immunity*.

[39] Rawlings N. D., Barrett A. J., Bateman A. (2010). MEROPS: the peptidase database. *Nucleic Acids Research*.

[40] Valdivieso E., Dagger F., Rascón A. (2007). _Leishmania mexicana_ : identification and characterization of an aspartyl proteinase activity. *Experimental Parasitology*.

[41] Sajid M., McKerrow J. H. (2002). Cysteine proteases of parasitic organisms. *Molecular and Biochemical Parasitology*.

[42] Vermelho A. B., Branquinha M. H., D’Ávila-Levy C. M., Souza dos Santos A. L., de Souza Dias E. P., Nogueira de Melo A. C. (2010). Biological roles of peptidases in trypanosomatids~!2009-11-26~!2010-02-15~!2010-03-18~!. *Open Parasitology Journal*.

[43] Frame M. J., Mottram J. C., Coombs G. H. (2000). Analysis of the roles of cysteine proteinases ofLeishmania mexicanain the host–parasite interaction. *Parasitology*.

[44] Davenport B. J., Martin C. G., Beverley S. M., Orlicky D. J., Vazquez-Torres A., Morrison T. E. (2018). SODB1 is essential for Leishmania major infection of macrophages and pathogenesis in mice. *PLoS Neglected Tropical Diseases*.

[45] Broxton C. N., Culotta V. C. (2016). SOD enzymes and microbial pathogens: surviving the oxidative storm of infection. *PLoS Pathogens*.

[46] Abreu I. A., Cabelli D. E. (2010). Superoxide dismutases--a review of the metal-associated mechanistic variations. *Biochimica et Biophysica Acta (BBA)-Proteins and Proteomics*.

[47] Butenko A., Kostygov A. Y., Sádlová J. (2019). Comparative genomics of Leishmania (Mundinia). *BMC Genomics*.

