# Supplementary material for: Genome Assembly and Genome Annotation of Leishmania martiniquensis Isolated from a Leishmaniasis Patient in Thailand
Source: J Parasitol Res. 2022 Mar 22;2022:8768574. doi: 10.1155/2022/8768574 (PMC8965598; doi:10.1155/2022/8768574)
Supplement: Supplementary 1 — Supplementary Material 1: the annotation lists of the candidate predicted virulence factor genes from ProtVirDB. [file 8768574.f1.pdf]

QueryID	Description
chr10_edit.fa.g105	Q4U925 Casein kinase II alpha (Establishment) [Theileria annulata]
chr10_edit.fa.g108	XP_001704890 CWP1 (Others) [Giardia duodenalis]
chr10_edit.fa.g114	XP_001350083 Rh2a (Adhesin) [Plasmodium falciparum]
chr10_edit.fa.g125	AAK49430 gp63 (Invasion) [Leishmania donovani]
chr10_edit.fa.g126	AAK49430 gp63 (Invasion) [Leishmania donovani]
chr10_edit.fa.g131	AAK49430 gp63 (Invasion) [Leishmania donovani]
chr10_edit.fa.g132	CAD42812 gp63 (Invasion) [Leishmania donovani]
chr10_edit.fa.g133	AAB30592 A2 (Others) [Leishmania donovani]
chr10_edit.fa.g136	AAK49430 gp63 (Invasion) [Leishmania donovani]
chr10_edit.fa.g137	AAK49430 gp63 (Invasion) [Leishmania donovani]
chr10_edit.fa.g138	AAK49430 gp63 (Invasion) [Leishmania donovani]
chr10_edit.fa.g139	AAK49430 gp63 (Invasion) [Leishmania donovani]
chr10_edit.fa.g140	AAK49430 gp63 (Invasion) [Leishmania donovani]
chr10_edit.fa.g142	AAK49430 gp63 (Invasion) [Leishmania donovani]
chr10_edit.fa.g144	AAK49430 gp63 (Invasion) [Leishmania donovani]
chr10_edit.fa.g146	AAA29237 gp63 (Invasion) [Leishmania donovani]
chr10_edit.fa.g147	AAK49430 gp63 (Invasion) [Leishmania donovani]
chr10_edit.fa.g149	AAK49430 gp63 (Invasion) [Leishmania donovani]
chr10_edit.fa.g152	CAB51784 gp63 (Invasion) [Leishmania donovani]
chr10_edit.fa.g153	CAB51787 gp63 (Invasion) [Leishmania donovani]
chr10_edit.fa.g155	CAB51785 gp63 (Invasion) [Leishmania donovani]
chr10_edit.fa.g157	P23223 gp63 (Invasion) [Leishmania donovani]
chr10_edit.fa.g158	CAB51786 gp63 (Invasion) [Leishmania donovani]
chr10_edit.fa.g54	Q4U925 Casein kinase II alpha (Establishment) [Theileria annulata]
chr10_edit.fa.g63	XP_001350083 Rh2a (Adhesin) [Plasmodium falciparum]
chr10_edit.fa.g72	XP_001350083 Rh2a (Adhesin) [Plasmodium falciparum]
chr10_edit.fa.g73	XP_652776 Rab11B (Establishment) [Entamoeba histolytica]
chr10_edit.fa.g74	XP_652776 Rab11B (Establishment) [Entamoeba histolytica]
chr10_edit.fa.g78	XP_652776 Rab11B (Establishment) [Entamoeba histolytica]
chr11_edit.fa.g170	XP_001350083 Rh2a (Adhesin) [Plasmodium falciparum]
chr11_edit.fa.g184	Q4U925 Casein kinase II alpha (Establishment) [Theileria annulata]
chr11_edit.fa.g189	AAP74702 TbVSP1 (Others) [Trypanosoma brucei]
chr11_edit.fa.g191	CAC43441 eIF4A (Others) [Toxoplasma gondii]

chr11_edit.fa.g199	Q4U925 Casein kinase II alpha (Establishment) [Theileria annulata]
chr11_edit.fa.g216	EAA36893 PAT (Others) [Giardia duodenalis]
chr11_edit.fa.g217	EAA36893 PAT (Others) [Giardia duodenalis]
chr11_edit.fa.g276	AAS67870 Leucyl aminopeptidase (Others) [Trypanosoma brucei]
chr12_edit.fa.g336	XP_001710242 CWP2 (Others) [Giardia duodenalis]
chr12_edit.fa.g388	XP_001350083 Rh2a (Adhesin) [Plasmodium falciparum]
chr12_edit.fa.g417	XP_001350083 Rh2a (Adhesin) [Plasmodium falciparum]
chr13_edit.fa.g456	CAC67416 Brucipain (Cysteine proteases) [Trypanosoma brucei]
chr13_edit.fa.g476	BAB40673 Rab5 (Establishment) [Entamoeba histolytica]
chr13_edit.fa.g523	Q4U925 Casein kinase II alpha (Establishment) [Theileria annulata]
chr13_edit.fa.g541	Q4U925 Casein kinase II alpha (Establishment) [Theileria annulata]
chr13_edit.fa.g554	XP_001350083 Rh2a (Adhesin) [Plasmodium falciparum]
chr13_edit.fa.g591	Q4U925 Casein kinase II alpha (Establishment) [Theileria annulata]
chr14_edit.fa.g608	EAA36893 PAT (Others) [Giardia duodenalis]
chr14_edit.fa.g656	XP_652776 Rab11B (Establishment) [Entamoeba histolytica]
chr14_edit.fa.g688	Q4U925 Casein kinase II alpha (Establishment) [Theileria annulata]
chr14_edit.fa.g699	Q4U925 Casein kinase II alpha (Establishment) [Theileria annulata]
chr14_edit.fa.g726	Q4U925 Casein kinase II alpha (Establishment) [Theileria annulata]
chr14_edit.fa.g740	XP_001350083 Rh2a (Adhesin) [Plasmodium falciparum]
chr15_edit.fa.g766	XP_001348165 RESA (Others) [plasmodium falciparum]
chr15_edit.fa.g801	Q4U925 Casein kinase II alpha (Establishment) [Theileria annulata]
chr15_edit.fa.g829	XP_001348165 RESA (Others) [plasmodium falciparum]
chr15_edit.fa.g833	Q4U925 Casein kinase II alpha (Establishment) [Theileria annulata]
chr15_edit.fa.g858	CAC43441 eIF4A (Others) [Toxoplasma gondii]
chr15_edit.fa.g863	Q4U925 Casein kinase II alpha (Establishment) [Theileria annulata]
chr15_edit.fa.g891	XP_820135 Trans-sialidase (Invasion) [Trypanosoma cruzi]
chr15_edit.fa.g900	XP_652003 Peroxiredoxin (Others) [Entamoeba histolytica]
chr15_edit.fa.g901	XP_652003 Peroxiredoxin (Others) [Entamoeba histolytica]
chr15_edit.fa.g907	XP_820135 Trans-sialidase (Invasion) [Trypanosoma cruzi]
chr16_edit.fa.g1128	AAL27793 CPSII (Establishment) [Toxoplasma gondii]
chr16_edit.fa.g1151	Q4U925 Casein kinase II alpha (Establishment) [Theileria annulata]
chr16_edit.fa.g1176	Q4U925 Casein kinase II alpha (Establishment) [Theileria annulata]
chr16_edit.fa.g928	AAC72001 Hsp70 (Heat shock protein) [Toxoplasma gondii]
chr16_edit.fa.g961	CAA87086 Hsp70 (Heat shock protein) [Eimeria tenella]

chr16_edit.fa.g996 Q05889 Galactofuranosyl glycosyltransferase (Others) [Leishmania donovani]
chr17_edit.fa.g1218 Q4U925 Casein kinase II alpha (Establishment) [Theileria annulata]
chr17_edit.fa.g1239 Q4U925 Casein kinase II alpha (Establishment) [Theileria annulata]
chr17_edit.fa.g1340 Q4U925 Casein kinase II alpha (Establishment) [Theileria annulata]
chr17_edit.fa.g1346 Q4U925 Casein kinase II alpha (Establishment) [Theileria annulata]
chr18_edit.fa.g1373 AAA29227 LDH1A (Establishment) [Leishmania donovani]
chr18_edit.fa.g1374 AAA29227 LDH1A (Establishment) [Leishmania donovani]
chr18_edit.fa.g1388 AAF75871 Hsp70 (Heat shock protein) [Cryptosporidium parvum]
chr18_edit.fa.g1396 XP_649196 Rab7A (Establishment) [Entamoeba histolytica]
chr18_edit.fa.g1424 XP_001682426 UDP-galactopyranose mutase (Others) [Leishmania donovani]
chr18_edit.fa.g1432 Q4U925 Casein kinase II alpha (Establishment) [Theileria annulata]
chr18_edit.fa.g1437 XP_652776 Rab11B (Establishment) [Entamoeba histolytica]
chr18_edit.fa.g1489 Q4U925 Casein kinase II alpha (Establishment) [Theileria annulata]
chr18_edit.fa.g1498 Q4U925 Casein kinase II alpha (Establishment) [Theileria annulata]
chr19_edit.fa.g1532 ABY64746 ADF (Invasion) [Eimeria tenella]
chr19_edit.fa.g1546 Q4U925 Casein kinase II alpha (Establishment) [Theileria annulata]
chr19_edit.fa.g1575 Q4U925 Casein kinase II alpha (Establishment) [Theileria annulata]
chr19_edit.fa.g1576 Q4U925 Casein kinase II alpha (Establishment) [Theileria annulata]
chr19_edit.fa.g1582 XP_001350083 Rh2a (Adhesin) [Plasmodium falciparum]
chr19_edit.fa.g1630 Q4U925 Casein kinase II alpha (Establishment) [Theileria annulata]
chr19_edit.fa.g1638 CAC67416 Brucipain (Cysteine proteases) [Trypanosoma brucei]
chr19_edit.fa.g1645 XP_001350083 Rh2a (Adhesin) [Plasmodium falciparum]
chr19_edit.fa.g1646 Q4U925 Casein kinase II alpha (Establishment) [Theileria annulata]
chr1_edit.fa.g1713 AAC72001 Hsp70 (Heat shock protein) [Toxoplasma gondii]
chr1_edit.fa.g1734 CAC43441 eIF4A (Others) [Toxoplasma gondii]
chr1_edit.fa.g1735 CAC43441 eIF4A (Others) [Toxoplasma gondii]
chr20_edit.fa.g1806 Q4U925 Casein kinase II alpha (Establishment) [Theileria annulata]
chr20_edit.fa.g1815 CAC43441 eIF4A (Others) [Toxoplasma gondii]
chr20_edit.fa.g1823 Q4U925 Casein kinase II alpha (Establishment) [Theileria annulata]
chr20_edit.fa.g1824 Q4U925 Casein kinase II alpha (Establishment) [Theileria annulata]
chr20_edit.fa.g1876 Q4U925 Casein kinase II alpha (Establishment) [Theileria annulata]
chr20_edit.fa.g1877 Q4U925 Casein kinase II alpha (Establishment) [Theileria annulata]
chr21_edit.fa.g1960 XP_652776 Rab11B (Establishment) [Entamoeba histolytica]
chr21_edit.fa.g1998 XP_001350083 Rh2a (Adhesin) [Plasmodium falciparum]

chr21_edit.fa.g2003 Q4U925 Casein kinase II alpha (Establishment) [Theileria annulata]
chr21_edit.fa.g2012 XP_001710242 CWP2 (Others) [Giardia duodenalis]
chr21_edit.fa.g2016 ABV49611 Thymidine kinase (Invasion) [Leishmania donovani]
chr21_edit.fa.g2037 EAA36893 PAT (Others) [Giardia duodenalis]
chr21_edit.fa.g2055 CAC43441 eIF4A (Others) [Toxoplasma gondii]
chr21_edit.fa.g2071 XP_652967 Vps35 (Establishment) [Entamoeba histolytica]
chr21_edit.fa.g2073 XP_001348165 RESA (Others) [plasmodium falciparum]
chr21_edit.fa.g2119 ABO14295 Calcineurin B (Invasion) [Trypanosoma cruzi]
chr21_edit.fa.g2121 Q4U925 Casein kinase II alpha (Establishment) [Theileria annulata]
chr21_edit.fa.g2126 Q4U925 Casein kinase II alpha (Establishment) [Theileria annulata]
chr21_edit.fa.g2146 Q4U925 Casein kinase II alpha (Establishment) [Theileria annulata]
chr22_edit.fa.g2211 Q4U925 Casein kinase II alpha (Establishment) [Theileria annulata]
chr22_edit.fa.g2246 EAA36893 PAT (Others) [Giardia duodenalis]
chr22_edit.fa.g2263 Q4U925 Casein kinase II alpha (Establishment) [Theileria annulata]
chr22_edit.fa.g2279 Q4U925 Casein kinase II alpha (Establishment) [Theileria annulata]
chr22_edit.fa.g2301 AAB30592 A2 (Others) [Leishmania donovani]
chr22_edit.fa.g2310 AAB30592 A2 (Others) [Leishmania donovani]
chr22_edit.fa.g2311 AAB30592 A2 (Others) [Leishmania donovani]
chr22_edit.fa.g2318 AAB30592 A2 (Others) [Leishmania donovani]
chr23_edit.fa.g2406 XP_652003 Peroxiredoxin (Others) [Entamoeba histolytica]
chr23_edit.fa.g2446 AAS67870 Leucyl aminopeptidase (Others) [Trypanosoma brucei]
chr23_edit.fa.g2495 EAA36893 PAT (Others) [Giardia duodenalis]
chr23_edit.fa.g2496 EAA36893 PAT (Others) [Giardia duodenalis]
chr23_edit.fa.g2503 EAA36893 PAT (Others) [Giardia duodenalis]
chr24_edit.fa.g2513 Q4U925 Casein kinase II alpha (Establishment) [Theileria annulata]
chr24_edit.fa.g2566 CAD68976 ICP (Others) [Trypanosoma brucei]
chr24_edit.fa.g2570 Q4U925 Casein kinase II alpha (Establishment) [Theileria annulata]
chr24_edit.fa.g2597 XP_001350083 Rh2a (Adhesin) [Plasmodium falciparum]
chr24_edit.fa.g2650 Q4U925 Casein kinase II alpha (Establishment) [Theileria annulata]
chr24_edit.fa.g2671 Q36736 KMP-11 (Others) [Leishmania donovani]
chr24_edit.fa.g2672 Q36736 KMP-11 (Others) [Leishmania donovani]
chr24_edit.fa.g2673 Q36736 KMP-11 (Others) [Leishmania donovani]
chr24_edit.fa.g2697 Q4U925 Casein kinase II alpha (Establishment) [Theileria annulata]
chr24_edit.fa.g2733 AAA87406 AP65-1 (Adhesin) [Trichomonas vaginalis]

chr24_edit.fa.g2735 AAA87406 AP65-1 (Adhesin) [Trichomonas vaginalis]
chr25_edit.fa.g2753 Q05889 Galactofuranosyl glycosyltransferase (Others) [Leishmania donovani]
chr25_edit.fa.g2843 AAC48339 AP33-3 (Adhesin) [Trichomonas vaginalis]
chr25_edit.fa.g2844 AAC48339 AP33-3 (Adhesin) [Trichomonas vaginalis]
chr25_edit.fa.g2859 BAB40673 Rab5 (Establishment) [Entamoeba histolytica]
chr25_edit.fa.g2901 Q4U925 Casein kinase II alpha (Establishment) [Theileria annulata]
chr25_edit.fa.g2915 EAA36893 PAT (Others) [Giardia duodenalis]
chr25_edit.fa.g2921 Q4U925 Casein kinase II alpha (Establishment) [Theileria annulata]
chr25_edit.fa.g2983 XP_001350083 Rh2a (Adhesin) [Plasmodium falciparum]
chr26_edit.fa.g3036 Q4U925 Casein kinase II alpha (Establishment) [Theileria annulata]
chr26_edit.fa.g3060 XP_001350083 Rh2a (Adhesin) [Plasmodium falciparum]
chr26_edit.fa.g3120 Q4U925 Casein kinase II alpha (Establishment) [Theileria annulata]
chr26_edit.fa.g3156 Q4U925 Casein kinase II alpha (Establishment) [Theileria annulata]
chr27_edit.fa.g3185 CAC43441 eIF4A (Others) [Toxoplasma gondii]
chr27_edit.fa.g3216 XP_001704890 CWP1 (Others) [Giardia duodenalis]
chr27_edit.fa.g3233 XP_001350083 Rh2a (Adhesin) [Plasmodium falciparum]
chr27_edit.fa.g3265 XP_001350083 Rh2a (Adhesin) [Plasmodium falciparum]
chr27_edit.fa.g3266 Q4U925 Casein kinase II alpha (Establishment) [Theileria annulata]
chr27_edit.fa.g3267 AAK69358 P0 (Invasion) [Toxoplasma gondii]
chr27_edit.fa.g3268 AAK69358 P0 (Invasion) [Toxoplasma gondii]
chr27_edit.fa.g3272 Q4U925 Casein kinase II alpha (Establishment) [Theileria annulata]
chr27_edit.fa.g3297 XP_652776 Rab11B (Establishment) [Entamoeba histolytica]
chr27_edit.fa.g3313 Q4U925 Casein kinase II alpha (Establishment) [Theileria annulata]
chr27_edit.fa.g3317 XP_001350083 Rh2a (Adhesin) [Plasmodium falciparum]
chr27_edit.fa.g3351 Q4U925 Casein kinase II alpha (Establishment) [Theileria annulata]
chr27_edit.fa.g3374 Q4U925 Casein kinase II alpha (Establishment) [Theileria annulata]
chr27_edit.fa.g3403 CAC43441 eIF4A (Others) [Toxoplasma gondii]
chr27_edit.fa.g3407 Q4U925 Casein kinase II alpha (Establishment) [Theileria annulata]
chr27_edit.fa.g3418 XP_808957 Trans-sialidase (Invasion) [Trypanosoma cruzi]
chr27_edit.fa.g3479 XP_001348165 RESA (Others) [plasmodium falciparum]
chr27_edit.fa.g3518 AAC80459 Oligopeptidase B (Others) [Trypanosoma brucei]
chr27_edit.fa.g3520 XP_001348153 MAEBL (Adhesin) [Plasmodium falciparum]
chr28_edit.fa.g3526 EAA36893 PAT (Others) [Giardia duodenalis]
chr28_edit.fa.g3547 Q4U925 Casein kinase II alpha (Establishment) [Theileria annulata]

chr28_edit.fa.g3563	CAC43441	eIF4A	(Others)	[Toxoplasma gondii]
chr28_edit.fa.g3604	AAC72001	Hsp70	(Heat shock protein)	[Toxoplasma gondii]
chr28_edit.fa.g3607	AAC72001	Hsp70	(Heat shock protein)	[Toxoplasma gondii]
chr28_edit.fa.g3622	Q4U925	Casein kinase II alpha	(Establishment)	[Theileria annulata]
chr28_edit.fa.g3644	XP_001350083	Rh2a	(Adhesin)	[Plasmodium falciparum]
chr28_edit.fa.g3677	CAC43441	eIF4A	(Others)	[Toxoplasma gondii]
chr28_edit.fa.g3689	AAC72001	Hsp70	(Heat shock protein)	[Toxoplasma gondii]
chr28_edit.fa.g3718	Q4U925	Casein kinase II alpha	(Establishment)	[Theileria annulata]
chr28_edit.fa.g3719	CAC43441	eIF4A	(Others)	[Toxoplasma gondii]
chr29_edit.fa.g3804	XP_001351221	CTRP	(Adhesin)	[Plasmodium falciparum]
chr29_edit.fa.g3812	XP_652776	Rab11B	(Establishment)	[Entamoeba histolytica]
chr29_edit.fa.g3822	Q4U925	Casein kinase II alpha	(Establishment)	[Theileria annulata]
chr29_edit.fa.g3864	Q4U925	Casein kinase II alpha	(Establishment)	[Theileria annulata]
chr29_edit.fa.g3884	Q4U925	Casein kinase II alpha	(Establishment)	[Theileria annulata]
chr29_edit.fa.g3901	Q4U925	Casein kinase II alpha	(Establishment)	[Theileria annulata]
chr29_edit.fa.g3921	Q4U925	Casein kinase II alpha	(Establishment)	[Theileria annulata]
chr29_edit.fa.g3934	BAB40673	Rab5	(Establishment)	[Entamoeba histolytica]
chr29_edit.fa.g3948	AAL87662	Dynamin-like protein	(Others)	[Giardia duodenalis]
chr29_edit.fa.g3959	Q4U925	Casein kinase II alpha	(Establishment)	[Theileria annulata]
chr29_edit.fa.g3969	AAM00390	LPG3	(Others)	[Leishmania donovani]
chr29_edit.fa.g3980	AAR88085	TbcatB	(Cysteine proteases)	[Trypanosoma brucei]
chr2_edit.fa.g4049	Q4U925	Casein kinase II alpha	(Establishment)	[Theileria annulata]
chr2_edit.fa.g4058	BAB40673	Rab5	(Establishment)	[Entamoeba histolytica]
chr2_edit.fa.g4062	Q4U925	Casein kinase II alpha	(Establishment)	[Theileria annulata]
chr2_edit.fa.g4069	Q4U925	Casein kinase II alpha	(Establishment)	[Theileria annulata]
chr30_edit.fa.g4085	Q4U925	Casein kinase II alpha	(Establishment)	[Theileria annulata]
chr30_edit.fa.g4098	XP_001350083	Rh2a	(Adhesin)	[Plasmodium falciparum]
chr30_edit.fa.g4109	Q4U925	Casein kinase II alpha	(Establishment)	[Theileria annulata]
chr30_edit.fa.g4127	Q4U925	Casein kinase II alpha	(Establishment)	[Theileria annulata]
chr30_edit.fa.g4131	XP_001350083	Rh2a	(Adhesin)	[Plasmodium falciparum]
chr30_edit.fa.g4176	BAB40673	Rab5	(Establishment)	[Entamoeba histolytica]
chr30_edit.fa.g4191	Q4U925	Casein kinase II alpha	(Establishment)	[Theileria annulata]
chr30_edit.fa.g4255	Q4U925	Casein kinase II alpha	(Establishment)	[Theileria annulata]
chr30_edit.fa.g4270	Q4U925	Casein kinase II alpha	(Establishment)	[Theileria annulata]

chr30_edit.fa.g4310 EAA36893 PAT (Others) [Giardia duodenalis]
chr30_edit.fa.g4380 XP_001350083 Rh2a (Adhesin) [Plasmodium falciparum]
chr30_edit.fa.g4387 Q4U925 Casein kinase II alpha (Establishment) [Theileria annulata]
chr30_edit.fa.g4441 XP_001350083 Rh2a (Adhesin) [Plasmodium falciparum]
chr30_edit.fa.g4442 CAA87086 Hsp70 (Heat shock protein) [Eimeria tenella]
chr31_edit.fa.g4466 EAA36893 PAT (Others) [Giardia duodenalis]
chr31_edit.fa.g4498 Q4U925 Casein kinase II alpha (Establishment) [Theileria annulata]
chr31_edit.fa.g4504 AAK49430 gp63 (Invasion) [Leishmania donovani]
chr31_edit.fa.g4505 Q4U925 Casein kinase II alpha (Establishment) [Theileria annulata]
chr31_edit.fa.g4509 Q4U925 Casein kinase II alpha (Establishment) [Theileria annulata]
chr31_edit.fa.g4571 XP_001710242 CWP2 (Others) [Giardia duodenalis]
chr31_edit.fa.g4573 XP_001704890 CWP1 (Others) [Giardia duodenalis]
chr31_edit.fa.g4574 XP_001704890 CWP1 (Others) [Giardia duodenalis]
chr31_edit.fa.g4575 XP_001704890 CWP1 (Others) [Giardia duodenalis]
chr31_edit.fa.g4641 AAK49430 gp63 (Invasion) [Leishmania donovani]
chr31_edit.fa.g4706 BAD34969 Rab7B (Establishment) [Entamoeba histolytica]
chr31_edit.fa.g4719 BAB40673 Rab5 (Establishment) [Entamoeba histolytica]
chr31_edit.fa.g4771 XP_001351221 CTRP (Adhesin) [Plasmodium falciparum]
chr32_edit.fa.g4811 AAP70314 Hemolysin (Invasion) [Leishmania donovani]
chr32_edit.fa.g4812 AAP70314 Hemolysin (Invasion) [Leishmania donovani]
chr32_edit.fa.g4820 XP_652937 Vps29 (Establishment) [Entamoeba histolytica]
chr32_edit.fa.g4879 BAB40673 Rab5 (Establishment) [Entamoeba histolytica]
chr32_edit.fa.g4882 Q4U925 Casein kinase II alpha (Establishment) [Theileria annulata]
chr32_edit.fa.g4941 Q4U925 Casein kinase II alpha (Establishment) [Theileria annulata]
chr32_edit.fa.g4955 CAC43441 eIF4A (Others) [Toxoplasma gondii]
chr32_edit.fa.g4971 Q4U925 Casein kinase II alpha (Establishment) [Theileria annulata]
chr32_edit.fa.g5046 Q05889 Galactofuranosyl glycosyltransferase (Others) [Leishmania donovani]
chr32_edit.fa.g5060 CAC43441 eIF4A (Others) [Toxoplasma gondii]
chr32_edit.fa.g5080 Q4U925 Casein kinase II alpha (Establishment) [Theileria annulata]
chr32_edit.fa.g5110 BAB40673 Rab5 (Establishment) [Entamoeba histolytica]
chr32_edit.fa.g5119 CAC43441 eIF4A (Others) [Toxoplasma gondii]
chr32_edit.fa.g5129 XP_001350083 Rh2a (Adhesin) [Plasmodium falciparum]
chr32_edit.fa.g5132 XP_652776 Rab11B (Establishment) [Entamoeba histolytica]
chr32_edit.fa.g5139 XP_001350083 Rh2a (Adhesin) [Plasmodium falciparum]

chr32_edit.fa.g5158 XP_652776 Rab11B (Establishment) [Entamoeba histolytica]
chr32_edit.fa.g5183 XP_001350083 Rh2a (Adhesin) [Plasmodium falciparum]
chr33_edit.fa.g5196 XP_001350083 Rh2a (Adhesin) [Plasmodium falciparum]
chr33_edit.fa.g5214 AAS67870 Leucyl aminopeptidase (Others) [Trypanosoma brucei]
chr33_edit.fa.g5231 XP_626924 Hsp90 (Heat shock protein) [Cryptosporidium parvum]
chr33_edit.fa.g5279 XP_001350083 Rh2a (Adhesin) [Plasmodium falciparum]
chr33_edit.fa.g5305 Q4U925 Casein kinase II alpha (Establishment) [Theileria annulata]
chr33_edit.fa.g5319 Q4U925 Casein kinase II alpha (Establishment) [Theileria annulata]
chr33_edit.fa.g5376 XP_652776 Rab11B (Establishment) [Entamoeba histolytica]
chr33_edit.fa.g5392 Q4U925 Casein kinase II alpha (Establishment) [Theileria annulata]
chr33_edit.fa.g5441 Q4U925 Casein kinase II alpha (Establishment) [Theileria annulata]
chr33_edit.fa.g5452 Q4U925 Casein kinase II alpha (Establishment) [Theileria annulata]
chr33_edit.fa.g5457 EAA36893 PAT (Others) [Giardia duodenalis]
chr33_edit.fa.g5494 Q4U925 Casein kinase II alpha (Establishment) [Theileria annulata]
chr33_edit.fa.g5501 XP_626924 Hsp90 (Heat shock protein) [Cryptosporidium parvum]
chr34_edit.fa.g5582 XP_001350083 Rh2a (Adhesin) [Plasmodium falciparum]
chr34_edit.fa.g5671 Q05889 Galactofuranosyl glycosyltransferase (Others) [Leishmania donovani]
chr34_edit.fa.g5672 Q05889 Galactofuranosyl glycosyltransferase (Others) [Leishmania donovani]
chr34_edit.fa.g5715 XP_627530 p23 (Adhesin) [Cryptosporidium parvum]
chr34_edit.fa.g5794 CAC43441 eIF4A (Others) [Toxoplasma gondii]
chr34_edit.fa.g5833 XP_001350083 Rh2a (Adhesin) [Plasmodium falciparum]
chr34_edit.fa.g5835 AAP74702 TbVSP1 (Others) [Trypanosoma brucei]
chr34_edit.fa.g5869 Q4U925 Casein kinase II alpha (Establishment) [Theileria annulata]
chr34_edit.fa.g5881 Q4U925 Casein kinase II alpha (Establishment) [Theileria annulata]
chr34_edit.fa.g5887 AAC46914 LPG2 (Others) [Leishmania donovani]
chr34_edit.fa.g5908 AAY40294 Pyroglutamyl peptidase I (Others) [Trypanosoma brucei]
chr35_edit.fa.g5971 Q4U925 Casein kinase II alpha (Establishment) [Theileria annulata]
chr35_edit.fa.g5973 CAC43441 eIF4A (Others) [Toxoplasma gondii]
chr35_edit.fa.g5975 Q4U925 Casein kinase II alpha (Establishment) [Theileria annulata]
chr35_edit.fa.g5996 XP_001350083 Rh2a (Adhesin) [Plasmodium falciparum]
chr35_edit.fa.g6017 XP_627530 p23 (Adhesin) [Cryptosporidium parvum]
chr35_edit.fa.g6058 Q4U925 Casein kinase II alpha (Establishment) [Theileria annulata]
chr35_edit.fa.g6071 Q4U925 Casein kinase II alpha (Establishment) [Theileria annulata]
chr35_edit.fa.g6082 XP_001350083 Rh2a (Adhesin) [Plasmodium falciparum]

chr35_edit.fa.g6089 XP_001350083 Rh2a (Adhesin) [Plasmodium falciparum]
chr35_edit.fa.g6096 AAB86482 RabA (Invasion) [Entamoeba histolytica]
chr35_edit.fa.g6119 CAC43441 eIF4A (Others) [Toxoplasma gondii]
chr35_edit.fa.g6130 XP_001710242 CWP2 (Others) [Giardia duodenalis]
chr35_edit.fa.g6171 XP_001350083 Rh2a (Adhesin) [Plasmodium falciparum]
chr35_edit.fa.g6187 EAA36893 PAT (Others) [Giardia duodenalis]
chr35_edit.fa.g6204 CAC43441 eIF4A (Others) [Toxoplasma gondii]
chr35_edit.fa.g6229 Q4U925 Casein kinase II alpha (Establishment) [Theileria annulata]
chr35_edit.fa.g6247 AAC72001 Hsp70 (Heat shock protein) [Toxoplasma gondii]
chr35_edit.fa.g6254 Q4U925 Casein kinase II alpha (Establishment) [Theileria annulata]
chr35_edit.fa.g6258 Q4U925 Casein kinase II alpha (Establishment) [Theileria annulata]
chr35_edit.fa.g6273 AAA29227 LDH1A (Establishment) [Leishmania donovani]
chr35_edit.fa.g6309 XP_001348165 RESA (Others) [plasmodium falciparum]
chr35_edit.fa.g6428 Q4U925 Casein kinase II alpha (Establishment) [Theileria annulata]
chr35_edit.fa.g6430 Q4U925 Casein kinase II alpha (Establishment) [Theileria annulata]
chr35_edit.fa.g6440 Q05889 Galactofuranosyl glycosyltransferase (Others) [Leishmania donovani]
chr35_edit.fa.g6447 XP_001704890 CWP1 (Others) [Giardia duodenalis]
chr35_edit.fa.g6450 XP_001710242 CWP2 (Others) [Giardia duodenalis]
chr35_edit.fa.g6451 XP_001704890 CWP1 (Others) [Giardia duodenalis]
chr35_edit.fa.g6454 XP_001704890 CWP1 (Others) [Giardia duodenalis]
chr35_edit.fa.g6476 XP_001351221 CTRP (Adhesin) [Plasmodium falciparum]
chr36_edit.fa.g6489 Q4U925 Casein kinase II alpha (Establishment) [Theileria annulata]
chr36_edit.fa.g6521 AAP33064 Vps26 (Establishment) [Entamoeba histolytica]
chr36_edit.fa.g6533 XP_001350083 Rh2a (Adhesin) [Plasmodium falciparum]
chr36_edit.fa.g6570 AAB68610 AP51-2 (Adhesin) [Trichomonas vaginalis]
chr36_edit.fa.g6629 XP_001609101 variant erythrocyte surface antigen-1, alpha subunit (Establishment) [Babesia bovis]
chr36_edit.fa.g6660 BAD34969 Rab7B (Establishment) [Entamoeba histolytica]
chr36_edit.fa.g6717 Q4U925 Casein kinase II alpha (Establishment) [Theileria annulata]
chr36_edit.fa.g6777 ABH04323 LPTP1 (Establishment) [Leishmania donovani]
chr36_edit.fa.g6779 Q4U925 Casein kinase II alpha (Establishment) [Theileria annulata]
chr36_edit.fa.g6824 EAA36893 PAT (Others) [Giardia duodenalis]
chr36_edit.fa.g6830 XP_001704890 CWP1 (Others) [Giardia duodenalis]
chr36_edit.fa.g6847 Q4U925 Casein kinase II alpha (Establishment) [Theileria annulata]
chr36_edit.fa.g6848 Q4U925 Casein kinase II alpha (Establishment) [Theileria annulata]

chr36_edit.fa.g6874 CAC43441 eIF4A (Others) [Toxoplasma gondii]
chr36_edit.fa.g6875 XP_001350083 Rh2a (Adhesin) [Plasmodium falciparum]
chr36_edit.fa.g6887 XP_001350083 Rh2a (Adhesin) [Plasmodium falciparum]
chr36_edit.fa.g6956 XP_001350083 Rh2a (Adhesin) [Plasmodium falciparum]
chr36_edit.fa.g6989 CAC43441 eIF4A (Others) [Toxoplasma gondii]
chr36_edit.fa.g6994 ABH04323 LPTP1 (Establishment) [Leishmania donovani]
chr36_edit.fa.g7036 Q4U925 Casein kinase II alpha (Establishment) [Theileria annulata]
chr36_edit.fa.g7048 AAK69358 P0 (Invasion) [Toxoplasma gondii]
chr36_edit.fa.g7068 CAC43441 eIF4A (Others) [Toxoplasma gondii]
chr36_edit.fa.g7069 CAC43441 eIF4A (Others) [Toxoplasma gondii]
chr36_edit.fa.g7132 Q4U925 Casein kinase II alpha (Establishment) [Theileria annulata]
chr36_edit.fa.g7154 BAB40673 Rab5 (Establishment) [Entamoeba histolytica]
chr36_edit.fa.g7155 XP_001348165 RESA (Others) [plasmodium falciparum]
chr36_edit.fa.g7159 Q4U925 Casein kinase II alpha (Establishment) [Theileria annulata]
chr36_edit.fa.g7202 Q4U925 Casein kinase II alpha (Establishment) [Theileria annulata]
chr36_edit.fa.g7230 XP_001350083 Rh2a (Adhesin) [Plasmodium falciparum]
chr36_edit.fa.g7241 AAC80459 Oligopeptidase B (Others) [Trypanosoma brucei]
chr36_edit.fa.g7265 Q4U925 Casein kinase II alpha (Establishment) [Theileria annulata]
chr36_edit.fa.g7268 Q4U925 Casein kinase II alpha (Establishment) [Theileria annulata]
chr36_edit.fa.g7272 Q4U925 Casein kinase II alpha (Establishment) [Theileria annulata]
chr36_edit.fa.g7277 XP_001348599 Aldolase (Others) [Plasmodium falciparum]
chr3_edit.fa.g7344 AAP74702 TbVSP1 (Others) [Trypanosoma brucei]
chr3_edit.fa.g7416 Q4U925 Casein kinase II alpha (Establishment) [Theileria annulata]
chr3_edit.fa.g7418 CAC43441 eIF4A (Others) [Toxoplasma gondii]
chr3_edit.fa.g7452 Q4U925 Casein kinase II alpha (Establishment) [Theileria annulata]
chr4_edit.fa.g7473 XP_652776 Rab11B (Establishment) [Entamoeba histolytica]
chr4_edit.fa.g7497 XP_001350083 Rh2a (Adhesin) [Plasmodium falciparum]
chr4_edit.fa.g7501 AAA29227 LDH1A (Establishment) [Leishmania donovani]
chr4_edit.fa.g7516 AAK31238 variable surface protein 21f (Establishment) [Giardia duodenalis]
chr4_edit.fa.g7517 AAK31250 variable surface protein IVh (Establishment) [Giardia duodenalis]
chr4_edit.fa.g7539 XP_001348165 RESA (Others) [plasmodium falciparum]
chr4_edit.fa.g7572 AAA29227 LDH1A (Establishment) [Leishmania donovani]
chr4_edit.fa.g7587 BAB40673 Rab5 (Establishment) [Entamoeba histolytica]
chr5_edit.fa.g7632 CAC43441 eIF4A (Others) [Toxoplasma gondii]

chr5_edit.fa.g7633	Q4U925 Casein kinase II alpha (Establishment) [Theileria annulata]
chr5_edit.fa.g7675	Q4U925 Casein kinase II alpha (Establishment) [Theileria annulata]
chr5_edit.fa.g7698	Q4U925 Casein kinase II alpha (Establishment) [Theileria annulata]
chr5_edit.fa.g7701	CAC43441 eIF4A (Others) [Toxoplasma gondii]
chr6_edit.fa.g7713	AAC80459 Oligopeptidase B (Others) [Trypanosoma brucei]
chr6_edit.fa.g7774	XP_652776 Rab11B (Establishment) [Entamoeba histolytica]
chr6_edit.fa.g7792	Q4U925 Casein kinase II alpha (Establishment) [Theileria annulata]
chr6_edit.fa.g7808	XP_001350083 Rh2a (Adhesin) [Plasmodium falciparum]
chr6_edit.fa.g7814	Q4U925 Casein kinase II alpha (Establishment) [Theileria annulata]
chr7_edit.fa.g7860	CAC43441 eIF4A (Others) [Toxoplasma gondii]
chr7_edit.fa.g7880	Q4U925 Casein kinase II alpha (Establishment) [Theileria annulata]
chr7_edit.fa.g7898	Q4U925 Casein kinase II alpha (Establishment) [Theileria annulata]
chr7_edit.fa.g7928	XP_652776 Rab11B (Establishment) [Entamoeba histolytica]
chr7_edit.fa.g7971	Q4U925 Casein kinase II alpha (Establishment) [Theileria annulata]
chr8_edit.fa.g8029	CAC43441 eIF4A (Others) [Toxoplasma gondii]
chr8_edit.fa.g8065	Q4U925 Casein kinase II alpha (Establishment) [Theileria annulata]
chr9_edit.fa.g8144	XP_649196 Rab7A (Establishment) [Entamoeba histolytica]
chr9_edit.fa.g8146	CAC43441 eIF4A (Others) [Toxoplasma gondii]
chr9_edit.fa.g8153	AAC80459 Oligopeptidase B (Others) [Trypanosoma brucei]
chr9_edit.fa.g8182	Q4U925 Casein kinase II alpha (Establishment) [Theileria annulata]
