# Supplementary material for: Genome Assembly and Genome Annotation of Leishmania martiniquensis Isolated from a Leishmaniasis Patient in Thailand
Source: J Parasitol Res. 2022 Mar 22;2022:8768574. doi: 10.1155/2022/8768574 (PMC8965598; doi:10.1155/2022/8768574)
Supplement: Supplementary 3 — Supplementary Material 3: the annotation lists of the unique genes between L. martiniquensis (LU_Lmar_1.0) and our L. martiniquensis genome. [file 8768574.f3.pdf]

The annotation lists of the unique genes in our *L. martiniquensis* genome

ID	Description
ABCA3	ATP-binding cassette protein subfamily A, member
ACYP1	Belongs to the acylphosphatase family
ALG2	Dolichyl-P-Man GDP-Man1GlcNAc2-PP-dolichyl alpha-1,3-mannosyltransferase
ATG8C.1	Belongs to the ATG8 family
CACTIN	Cactus-binding C-terminus of cactin protein
CCBL2	Aminotransferase class-V
CYCS	Electron carrier protein. The oxidized form of the cytochrome c heme group can accept an electron from the heme group of the cytochrome c1 subunit of cytochrome reductase. Cytochrome c then transfers this electron to the cytochrome oxidase complex, the final protein carrier in the mitochondrial electron-transport chain
DKC1	centromere microtubule binding protein cbf5
dnajc16	DnaJ molecular chaperone homology domain
ERCC5	DNA repair protein RAD2
ERGIC3	Endoplasmic Reticulum-Golgi Intermediate Compartment (ERGIC)
GP63-2	Gene with more than two copies in the genome that does not belong to a merged region and that could not be assigned to a particular haplotype
GP63-3	Gene with more than two copies in the genome that does not belong to a merged region and that could not be assigned to a particular haplotype
H2AFB3	Histone H2A
HEXBP	Universal minicircle sequence binding protein (UMSBP)
HSP70.4	Belongs to the heat shock protein 70 family
IFT22	Ras of Complex, Roc, domain of DAPkinase
IKS1	Protein kinase, putative
INPP5E	endonuclease exonuclease phosphatase
ISA2	protein maturation by iron-sulfur cluster transfer
LEPP12	Phosphoprotein lepp12
LMAN1	Legume-like lectin family
malA	malic enzyme
MDH1	Malate dehydrogenase
MRPL46	39S mitochondrial ribosomal protein L46

The annotation lists of the unique genes in our *L. martiniquensis* genome (Conc.)

ID	Description
MSH4	Mis-match repair protein
MSRB3	SelR domain
NAPG	soluble NSF attachment protein activity
NIP7	Required for proper 27S pre-rRNA processing and 60S ribosome subunit assembly
POL30	This protein is an auxiliary protein of DNA polymerase delta and is involved in the control of eukaryotic DNA replication by increasing the polymerase's processibility during elongation of the leading strand
PSMA7	The proteasome is a multicatalytic proteinase complex which is characterized by its ability to cleave peptides with Arg, Phe, Tyr, Leu, and Glu adjacent to the leaving group at neutral or slightly basic pH
PUF9A	pumilio protein 9
PUF9B	pumilio protein 9
RNHii	Endonuclease that specifically degrades the RNA of RNA- DNA hybrids
RPL19	60S ribosomal protein L19
rpl24	structural constituent of ribosome
rps20	ribosomal protein S20
RPS21	40S ribosomal protein S21
RPS30	40S ribosomal protein S30
RPS3A	Belongs to the eukaryotic ribosomal protein eS1 family
SCG7	Phosphoglycan beta 1,3 galactosyltransferase
SCGR6	Phosphoglycan beta 1,3 galactosyltransferase
SER33	D-3-phosphoglycerate dehydrogenase-like protein
SODB1	Destroys radicals which are normally produced within the cells and which are toxic to biological systems
SRP54	Binds to the signal sequence of presecretory protein when they emerge from the ribosomes and transfers them to TRAM (translocating chain-associating membrane protein)
STX19	Syntaxin
TBCK	Domain in Tre-2, BUB2p, and Cdc16p. Probable Rab-GAPs.
TSR1	Protein of unknown function (DUF663)
UBC	Polyubiquitin
ZTA1	quinone oxidoreductase

The annotation lists of the unique genes in *L. martiniquensis* (LU_Lmar_1.0)

ID	Description
ABT1	RNA recognition motif
ANAPC11	anaphase promoting complex subunit protein
C9orf85	Uncharacterized conserved protein (DUF2039)
LMAN2L	Legume-like lectin family
LSM7	snRNP Sm proteins
rai1	DNA binding
RBX1	Ring-box protein 1
RPL39	60S ribosomal protein L39
RPS29	Ribosomal protein S29
SLC25A34	Belongs to the mitochondrial carrier (TC 2.A.29) family
