# Supplementary material for: Genome Assembly and Genome Annotation of Leishmania martiniquensis Isolated from a Leishmaniasis Patient in Thailand
Source: J Parasitol Res. 2022 Mar 22;2022:8768574. doi: 10.1155/2022/8768574 (PMC8965598; doi:10.1155/2022/8768574)
Supplement: Supplementary 4 — Supplementary Table 1: comparison of percent identity in Leishmania spp. including L. infantum, L. donovani, L. major Friedlin, L. Mexicana, L. braziliensis, L. martiniquensis, and our L. martiniquensis. Supplementary Table 2: comparison of functional category of putative protein-coding genes in Leishmania spp. genome. The alphabet A-G represent names of Leishmania species including L. martiniquensis (A), L. infantum (B), L. donovani (C), L. braziliensis (D), L. major strain Friedlin (E), L. mexicana (F), and L. martiniquensis (LU_Lmar_1.0) (G). [file 8768574.f4.docx]

**Supplementary Table 1** Comparison of percent identity in *Leishmania spp.* including *L. infantum*, *L. donovani*, *L. major Friedlin*, *L. Mexicana*, *L. braziliensis*, *L. martiniquensis* and our *L. martiniquensis* LU_Lmar_1.0.

| **Chr** | ***L. braziliensis*** | ***L. donovani*** | ***L. infantum*** | ***L. major*** | ***L. mexicana*** | ***L. martiniquensis*** |
| --- | --- | --- | --- | --- | --- | --- |
| Chr1 | 18.82 | 19.01 | 19.18 | 19.55 | 19.20 | 19.13 |
| Chr2 | 19.09 | 18.73 | 21.53 | 21.71 | 18.87 | 38.23 |
| Chr3 | 18.63 | 18.91 | 19.13 | 19.38 | 19.20 | 57.50 |
| Chr4 | 18.16 | 19.33 | 18.41 | 18.47 | 18.57 | 32.05 |
| Chr5 | 18.14 | 18.48 | 18.71 | 18.56 | 18.62 | 19.46 |
| Chr6 | 18.34 | 18.68 | 18.78 | 18.71 | 18.94 | 45.44 |
| Chr7 | 18.32 | 18.40 | 19.06 | 18.62 | 18.66 | 26.46 |
| Chr8 | 18.51 | 17.87 | 18.69 | 18.75 | 18.70 | 25.95 |
| Chr9 | 19.89 | 18.17 | 18.99 | 19.77 | 19.55 | 28.77 |
| Chr10 | 19.35 | 20.06 | 20.71 | 20.03 | 21.45 | 26.58 |
| Chr11 | 18.50 | 17.26 | 18.13 | 18.05 | 18.13 | 19.88 |
| Chr12 | 18.12 | 16.82 | 18.32 | 18.58 | 18.63 | 29.20 |
| Chr13 | 18.27 | 17.85 | 18.29 | 20.11 | 20.24 | 25.00 |
| Chr14 | 18.88 | 19.38 | 19.92 | 19.13 | 19.23 | 59.70 |
| Chr15 | 18.30 | 18.41 | 18.29 | 18.44 | 18.29 | 20.46 |
| Chr16 | 18.26 | 17.88 | 18.98 | 18.44 | 18.54 | 26.26 |
| Chr17 | 18.17 | 18.14 | 18.46 | 18.40 | 18.41 | 40.39 |
| Chr18 | 18.08 | 17.86 | 18.03 | 17.95 | 17.89 | 23.20 |
| Chr19 | 17.89 | 17.41 | 18.96 | 19.17 | 18.01 | 22.55 |
| Chr20 | 18.13 | 20.59 | 19.79 | 20.22 | 19.51 | 57.58 |
| Chr21 | 18.64 | 18.47 | 18.58 | 19.79 | 19.84 | 42.76 |
| Chr22 | 17.92 | 17.89 | 18.17 | 18.40 | 18.17 | 20.74 |
| Chr23 | 17.83 | 18.16 | 18.43 | 18.49 | 18.66 | 24.29 |
| Chr24 | 18.18 | 17.98 | 18.25 | 18.21 | 18.19 | 24.57 |
| Chr25 | 18.14 | 19.36 | 19.51 | 19.63 | 19.43 | 33.01 |
| Chr26 | 17.98 | 18.42 | 18.69 | 18.80 | 18.44 | 23.42 |
| Chr27 | 17.84 | 16.95 | 18.19 | 18.06 | 18.20 | 19.23 |
| Chr28 | 17.89 | 17.84 | 18.18 | 18.11 | 18.20 | 25.06 |
| Chr29 | 17.81 | 17.29 | 17.83 | 17.82 | 17.80 | 24.91 |
| Chr30 | 17.76 | 17.52 | 17.88 | 17.88 | 17.86 | 29.76 |
| Chr31 | 17.78 | 18.30 | 18.08 | 18.42 | 17.88 | 23.19 |
| Chr32 | 17.63 | 17.76 | 18.01 | 17.94 | 17.80 | 22.23 |
| Chr33 | 17.67 | 17.52 | 18.21 | 18.07 | 18.04 | 23.79 |
| Chr34 | 18.17 | 17.85 | 17.38 | 17.86 | 18.72 | 29.40 |
| Chr35 | 17.54 | 17.04 | 17.81 | 17.86 | NA | 20.76 |
| Chr36 | 17.53 | 17.46 | 18.60 | 17.77 | NA | 19.22 |

*Not Available (NA)

**Supplementary Table 2** Comparison of functional category of putative protein-coding genes in *Leishmania spp.* genome. The alphabet A-G represent name of *Leishmania* species including *L. martiniquensis* (A*)*, *L. infantum* (B), *L. donovani* (C), *L. braziliensis* (D), *L. major* strain Friedlin (E), *L. mexicana* (F) and *L. martiniquensis* LU_Lmar_1.0 (G*)*.

| **Functional category based on COG** | | **Number of genes in *Leishmania spp.*** | | | | | | |
| --- | --- | --- | --- | --- | --- | --- | --- | --- |
|  |  | A | B | C | D | E | F | G |
| *Information storage and processing* | |  |  |  |  |  |  |  |
| J | Translation, ribosomal structure, and biogenesis | 374 | 391 | 347 | 383 | 392 | 392 | 376 |
| A | RNA processing and modification | 227 | 231 | 227 | 224 | 229 | 230 | 224 |
| K | Transcription | 83 | 82 | 81 | 81 | 85 | 82 | 78 |
| L | Replication, recombination, and repair | 156 | 153 | 152 | 150 | 153 | 150 | 146 |
| B | Chromatin structure and dynamics | 45 | 50 | 45 | 59 | 55 | 54 | 49 |
| *Cellular processes and signaling* | |  |  |  |  |  |  |  |
| D | Cell cycle control, cell division, chromosome partitioning | 59 | 59 | 59 | 60 | 60 | 61 | 59 |
| Y | Nuclear structure | 3 | 3 | 3 | 3 | 3 | 3 | 3 |
| V | Defense mechanisms | 33 | 35 | 34 | 29 | 32 | 33 | 32 |
| T | Signal transduction mechanisms | 317 | 313 | 307 | 306 | 309 | 309 | 290 |
| M | Cell wall/membrane/envelope biogenesis | 15 | 14 | 14 | 16 | 15 | 15 | 13 |
| N | Cell motility | 13 | 14 | 11 | 13 | 14 | 14 | 13 |
| Z | Cytoskeleton | 148 | 143 | 139 | 149 | 172 | 151 | 146 |
| W | Extracellular structures | 2 | 2 | 2 | 2 | 2 | 2 | 2 |
| U | Intracellular trafficking, secretion, and vesicular transport | 218 | 227 | 223 | 212 | 219 | 221 | 208 |
| O | Posttranslational modification, protein turnover, chaperones | 404 | 427 | 412 | 436 | 452 | 429 | 425 |
| *Metabolism* | |  |  |  |  |  |  |  |
| C | Energy production and conversion | 150 | 155 | 152 | 155 | 159 | 155 | 132 |
| G | Carbohydrate transport and metabolism | 219 | 200 | 186 | 224 | 212 | 198 | 190 |
| E | Amino acid transport and metabolism | 168 | 166 | 159 | 163 | 164 | 160 | 144 |
| F | Nucleotide transport and metabolism | 74 | 72 | 71 | 68 | 68 | 69 | 73 |
| H | Coenzyme transport and metabolism | 134 | 133 | 132 | 126 | 129 | 131 | 134 |
| I | Lipid transport and metabolism | 203 | 201 | 198 | 198 | 204 | 200 | 176 |
| P | Inorganic ion transport and metabolism | 87 | 94 | 87 | 87 | 87 | 88 | 84 |
| Q | Secondary metabolites biosynthesis, transport, and catabolism | 100 | 87 | 87 | 89 | 95 | 90 | 89 |
| *Poorly characterized* | |  |  |  |  |  |  |  |
| S | Function unknown | 1515 | 1530 | 1480 | 1521 | 1636 | 1559 | 1429 |
