# Supplementary material for: Genome Assembly and Genome Annotation of Leishmania martiniquensis Isolated from a Leishmaniasis Patient in Thailand
Source: J Parasitol Res. 2022 Mar 22;2022:8768574. doi: 10.1155/2022/8768574 (PMC8965598; doi:10.1155/2022/8768574)
Supplement: Supplementary 5 — Supplementary Figure 1: the functional annotation of L. martiniquensis. Supplementary Figure 2: the COG functional analysis of candidate virulence factor protein-coding genes. [file 8768574.f5.docx]

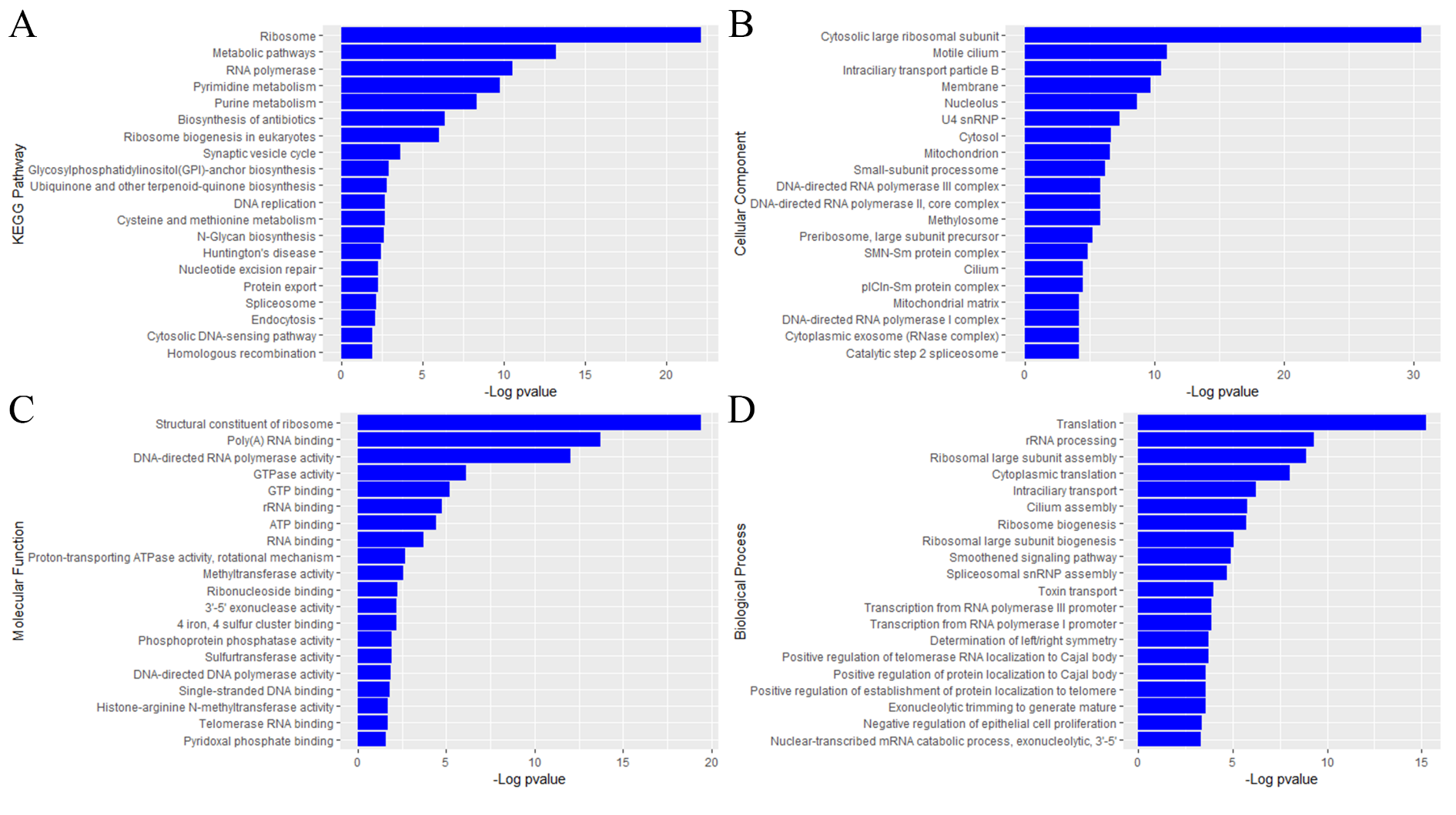


**Supplementary Figure 1** The functional annotation of *L. martiniquensis*. (A) The KEGG pathway annotation, (B) The cellular component annotation, (C) The molecular function annotation, and (D) The biological process annotation.


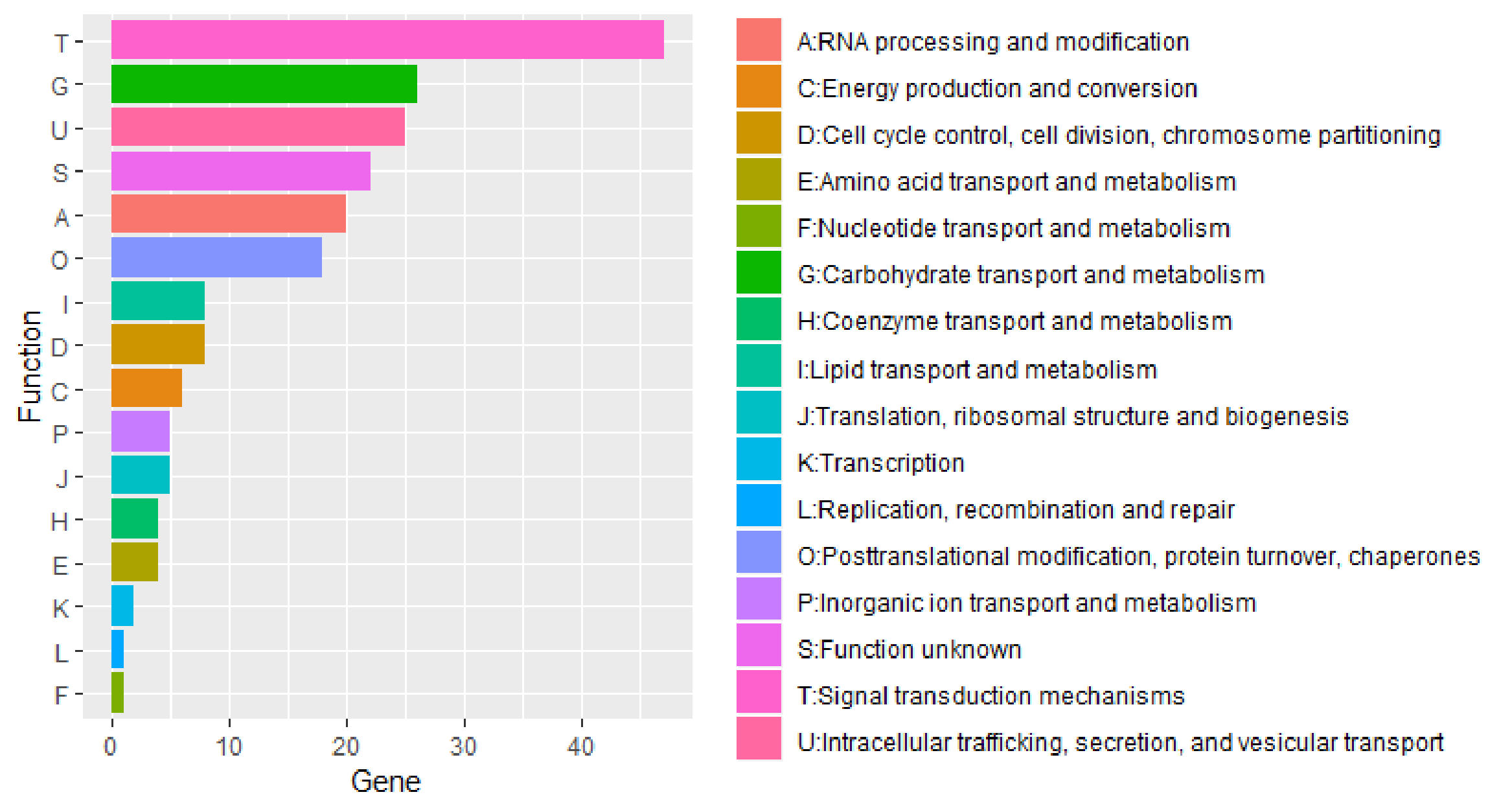


**Supplementary Figure 2** The COG functional analysis of candidate virulence factor protein-coding genes.
